# Global Longitudinal Strain and Biomarker Dynamics in Children With Acute Rheumatic Fever and Mitral Regurgitation: A 12-Month Prospective Study

**DOI:** 10.7759/cureus.97492

**Published:** 2025-11-22

**Authors:** Serkan F Çelik, Ugur Karagoz

**Affiliations:** 1 Pediatric Cardiology, Adnan Menderes University, Aydın, TUR; 2 Pediatric Cardiac Surgery, Adnan Menderes University, Aydın, TUR

**Keywords:** acute rheumatic fever, echocardiography, global longitudinal strain, mitral regurgitation, nt-probnp

## Abstract

Background: Acute rheumatic fever (ARF) remains a significant cause of acquired heart disease in children, particularly in developing countries. Although left ventricular systolic function is usually preserved, subclinical myocardial dysfunction may be present. Global longitudinal strain (GLS) is a sensitive parameter for detecting early myocardial impairment.

Objectives: This study aimed to evaluate subclinical myocardial dysfunction in children with ARF-related carditis using GLS and other strain parameters and to assess its progression over a one-year follow-up period according to the severity of mitral regurgitation (MR).

Methods: A total of 30 children with ARF-related carditis and preserved systolic function were prospectively enrolled. Patients were divided into two subgroups based on MR severity: moderate MR (Group 1, n = 12) and mild MR (Group 2, n = 18). Conventional echocardiographic parameters and speckle-tracking-derived strain measures were assessed at diagnosis, six months, and 12 months. GLS values were compared with those of age- and sex-matched healthy controls (n = 52).

Results: At diagnosis, GLS was significantly lower in ARF patients than controls (-19.9 ± 2.86 vs. -22.6 ± 4.65; p = 0.02) and at six months (-20.4 ± 3.42 vs. -22.6 ± 4.65, p = 0.04). Although GLS improved in the mild MR subgroup (-21.9 ± 3.82 vs. -22.6 ± 4.65, p = 0.41), it remained significantly impaired in the moderate MR subgroup (-19.25 ± 3.32 vs. -22.6 ± 4.65, p < 0.01) at 12 months. In multivariable linear regression analysis, GLS was negatively correlated with the severity of valve regurgitation (r = -0.53, p = 0.02) and NT-proBNP (r = -0.62, p = 0.012).

Conclusion: GLS and NT-proBNP are sensitive indicators of subclinical myocardial dysfunction in children with ARF-related carditis, especially in those with moderate MR. Longitudinal strain imaging may enhance early detection and guide long-term monitoring.

## Introduction

Acute rheumatic fever (ARF) is a post-infectious inflammatory disease following group A streptococcal pharyngitis and remains a major cause of acquired heart disease in children worldwide [1]. It can affect the heart (rheumatic carditis), joints, skin, and central nervous system and may progress to chronic rheumatic heart disease (RHD) with permanent valvular damage, particularly of the mitral valve [2].

Although ARF and RHD have long been considered primarily valvular disorders, recent evidence indicates that the myocardium may also be affected during the acute phase. Conventional echocardiography often fails to detect subtle myocardial dysfunction when the left ventricular ejection fraction is preserved. In this context, speckle-tracking echocardiography (STE) and especially global longitudinal strain (GLS) have gained attention as sensitive tools for detecting subclinical myocardial involvement [3,4].

Previous studies have shown impaired strain values in patients with valvular disease and chronic RHD despite normal systolic function [5-8]. However, data on myocardial strain specifically during the acute phase of ARF are still limited. Additionally, it remains unclear whether these early myocardial abnormalities resolve or persist during follow-up.

Therefore, this prospective study aimed to assess myocardial strain in children with ARF from diagnosis through a 12-month follow-up period to determine whether STE can detect ongoing or reversible myocardial involvement. Also, both GLS and NT-proBNP were assessed as complementary markers of subclinical myocardial dysfunction, reflecting mechanical deformation and biochemical stress, respectively.

## Materials and methods

This prospective observational study was conducted at a university hospital's pediatric cardiology department. Children aged 5-18 years diagnosed with ARF according to the Jones criteria were included if they had normal systolic function and no congenital heart disease, active pulmonary disease, chronic renal insufficiency, or diabetes mellitus. Patients receiving corticosteroids instead of salicylates were excluded. Patients with severe mitral regurgitation (MR) were excluded because significant volume overload and altered loading conditions could affect myocardial strain values and confound the assessment of subclinical myocardial involvement.

The control group consisted of 52 age- and sex-matched healthy children who were referred for non-cardiac evaluations, had normal echocardiographic findings, had no history of ARF, had no chronic inflammatory or cardiac disease, and had not received anti-inflammatory or cardiotoxic medications within the prior three months.

Blood samples were collected to analyze biomarkers of inflammation, myocardial injury, and stress, including cardiac troponin I (cTnI), NT-proBNP, and high-sensitivity CRP, using immunochromatographic methods. Only patients with carditis were included. Carditis was defined by valve annulus dilation (z-score > +2 adjusted for body surface area and age) or ≥50% reduction in diastolic leaflet excursion, pericardial effusion ≥5 mm, or mitral/aortic regurgitation [9]. MR severity was determined by color Doppler echocardiography relative to left atrial jet area: mild <20%, moderate 20-40%, and severe >40% [10].

Detailed medical histories from parents and recent medical records were reviewed. Fever was defined as an axillary temperature >38 °C and accepted as a major clinical criterion. ARF was treated with benzathine benzylpenicillin G, salicylates, or corticosteroids when indicated. Echocardiographic assessment was performed at diagnosis and repeated 12-16 weeks after treatment.

All echocardiographic examinations were performed by an experienced pediatric cardiologist using a GE VIVID S70 (GE Healthcare, Chicago, IL, USA). Standard measurements included LVIDd, LVIDs, LAd, fractional shortening (FS), and ejection fraction (EF) assessed by the Simpson method [11].

The severity of MR was graded according to the American Society of Echocardiography (ASE) pediatric guidelines, incorporating multiple parameters, including jet area relative to left atrial size, vena contracta width, flow reversal in pulmonary veins, and mitral annular dilation and leaflet morphology [9].

STE was performed according to current guidelines using grayscale images (60-100 fps) from apical and short-axis views. Endocardial borders were manually traced, and epicardial contours were auto-generated and adjusted if necessary. GLS was calculated as the average of six basal and six mid-ventricular segments [12,13].

To minimize bias, all image acquisitions and strain analyses were performed by a single pediatric cardiologist blinded to laboratory results and clinical subgrouping, and statistical analyses were conducted by an independent, blinded statistician. Although full double-blinding was not feasible in this clinical setting, observer bias was minimized by ensuring that the cardiologist performing strain measurements was blinded to NT-proBNP levels, clinical severity (mild vs. moderate MR), and follow-up timing. In addition, inter-observer variability was avoided by having all strain analyses performed by a single experienced observer using the same software and standardized acquisition protocol.

A linear mixed-effects model with random intercepts at the patient level was used to evaluate repeated measures, including fixed effects for time, MR group, and their interaction. NT-proBNP was log10-transformed; multiplicity was adjusted using the Benjamini-Hochberg false discovery rate. Bonferroni or Holm-Bonferroni corrections were applied to post hoc comparisons. A p-value < 0.05 was considered statistically significant. Analyses were performed using SPSS Inc. Released 2008. SPSS Statistics for Windows, Version 17.0 (SPSS Inc., Chicago, IL, USA).

Subgroup classification

Patients were stratified into two subgroups according to the severity of valve regurgitation at baseline as follows: Group 1: patients with moderate MR, and Group 2: patients with mild MR.

Statistical analysis

Continuous variables were expressed as mean ± standard deviation or median (interquartile range), and categorical variables as frequency (percentage). Group comparisons were performed using the independent-samples t-test or Mann-Whitney U test for continuous data, and the χ² test or Fisher's exact test for categorical variables, as appropriate.

To evaluate longitudinal changes and group-time interactions, a linear mixed-effects model with random intercepts for subjects was used across three time points (baseline, six months, and 12 months). The model included fixed effects for time, MR severity group (mild vs. moderate), and the time × group interaction and was adjusted for age, sex, and baseline left ventricular end-diastolic diameter. Missing data were handled using the restricted maximum likelihood (REML) method. Model outcomes were reported using mean differences, 95% confidence intervals (CIs), and exact p-values.

Where significant main or interaction effects were detected, Bonferroni-adjusted post hoc comparisons were performed, and findings were cross-validated using Holm-Bonferroni correction to avoid over-conservatism. At each time point, the correlation between GLS (absolute values, %) and log10-transformed NT-proBNP was assessed using two-tailed Pearson correlation analyses, separately in the carditis and control groups.

Additionally, to estimate effect sizes, multivariable linear regression analyses were conducted using NT-proBNP as the dependent variable and GLS and MR severity as predictors, with β-coefficients, 95% CIs, and false discovery rate (FDR)-adjusted p-values reported according to the Benjamini-Hochberg procedure. A two-tailed p-value < 0.05 was considered statistically significant. All statistical analyses were conducted using SPSS Inc. Released 2008. SPSS Statistics for Windows, Version 17.0 (SPSS Inc., Chicago, IL, USA).

## Results

After exclusions, 30 children with ARF-related carditis (mean age = 13.1 ± 4.1 years; 33% female) and 52 healthy controls (mean age = 13.8 ± 5.1 years; 40% female) were included. The two groups were comparable in age, sex distribution, and body mass index (p > 0.05 for all).

Fever was the most common major manifestation, observed in 24 patients (80%), followed by arthralgia in 22 patients (73%), arthritis in 15 patients (50%), and chorea or erythema marginatum in one patient each (3%). A prolonged PR interval was detected in 12 patients (39%). MR was present in 28 patients (93%), of whom 16 (53%) had mild MR and 12 (40%) had moderate MR. Eight children (27%) also exhibited aortic regurgitation, which was generally mild in degree. No patient demonstrated severe valvular dysfunction, and all showed preserved systolic function on echocardiography.

Conventional echocardiographic parameters, including EF, FS, left ventricular end-systolic diameter (LVIDs), left ventricular end-diastolic diameter (LVIDd), left ventricular posterior wall thickness (LVPWd), and left atrial diameter (LAd), were comparable between groups at baseline. At diagnosis, mean EF was 60.4 ± 2.9% in patients versus 63.6 ± 3.9% in controls (p = 0.25), and FS was 30.2 ± 2.1% in both groups (p = 0.42). LVIDs and LVIDd were slightly higher in patients (20.9 ± 2.4 mm/m² and 35.1 ± 4.8 mm/m², respectively), but these differences were not significant (p > 0.05). LVPWd and LAd values were also similar between groups (p > 0.05). During follow-up, minor nonsignificant increases were noted in LV dimensions and EF. At six months, mean EF was 62.8 ± 3.9%, and by 12 months, 62.5 ± 3.8%, showing a modest upward trend without statistical significance (p = 0.08). Similarly, FS remained stable (30.6 ± 2.1% at six months and 30.7 ± 2.2% at 12 months). No significant longitudinal changes were observed in LVIDd, LVIDs, LVPWd, or LAd over the 12-month period (p > 0.05 for all). These findings confirm that conventional echocardiographic indices of systolic function were preserved throughout follow-up.

GLS was significantly reduced in ARF patients compared with controls at diagnosis (-19.9 ± 2.86 vs. -22.6 ± 4.65; p = 0.02) and remained lower at six months (-20.4 ± 3.42 vs. -22.6 ± 4.65; p = 0.04). At 12 months, GLS improved (-21.4 ± 3.63; p = 0.38), approaching control values. Segmental strain values, including GLS-4C, GLS-3C, and GLS-2C, did not differ significantly between groups at any time point (all p > 0.05). Similarly, circumferential (-17.1 ± 3.8 vs. -17.6 ± 4.6; p > 0.05) and radial strain (54.1 ± 11.4 vs. 55.4 ± 11.8; p > 0.05) parameters showed no significant differences over time.

**Figure 1:**
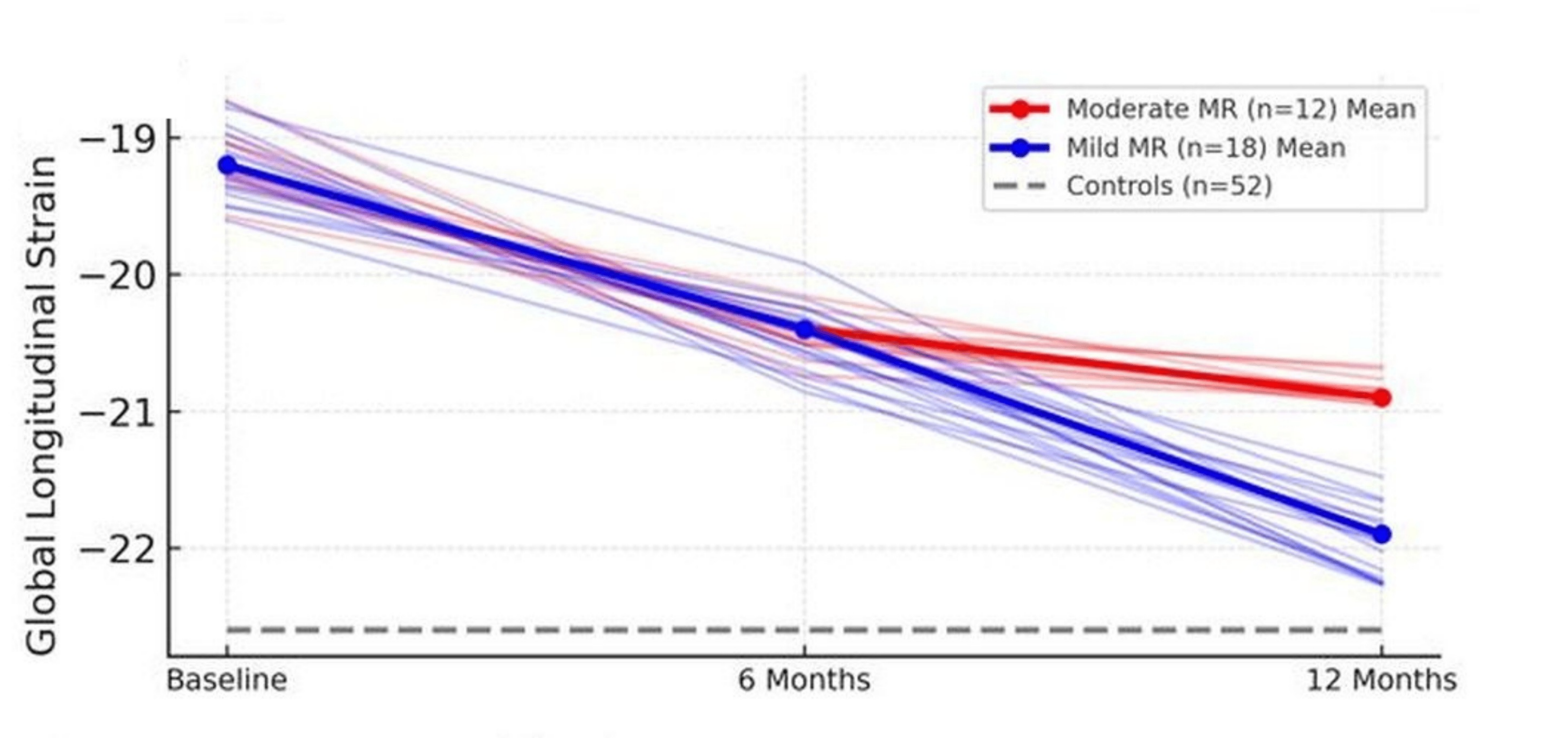
Global longitudinal strain (GLS, absolute %) across baseline, six months, and 12 months in controls and patients stratified by mitral regurgitation (MR) severity. The thin "spaghetti" lines show individual trajectories, and bold lines indicate group means. The control mean is shown as a gray-dashed reference line. Data are expressed as mean ± SD. Group comparisons and temporal changes were analyzed using repeated-measures ANOVA with Bonferroni correction. Abbreviations: GLS = global longitudinal strain; MR = mitral regurgitation.

As illustrated in Figure 1, GLS values progressively improved over 12 months but remained lower in the moderate MR subgroup. These findings suggest that GLS is a more sensitive indicator of subclinical myocardial dysfunction than segmental or circumferential strain indices. At baseline, NT-proBNP, CRP, and cTnI levels were markedly elevated (p < 0.001 for all) and declined significantly over time (p < 0.001, time effect). By six months, NT-proBNP and cTnI had decreased substantially, and CRP was near normal. At 12 months, CRP and cTnI had normalized (p > 0.05 vs. controls), while NT-proBNP remained mildly elevated in the carditis group (p < 0.05).

**Figure 2:**
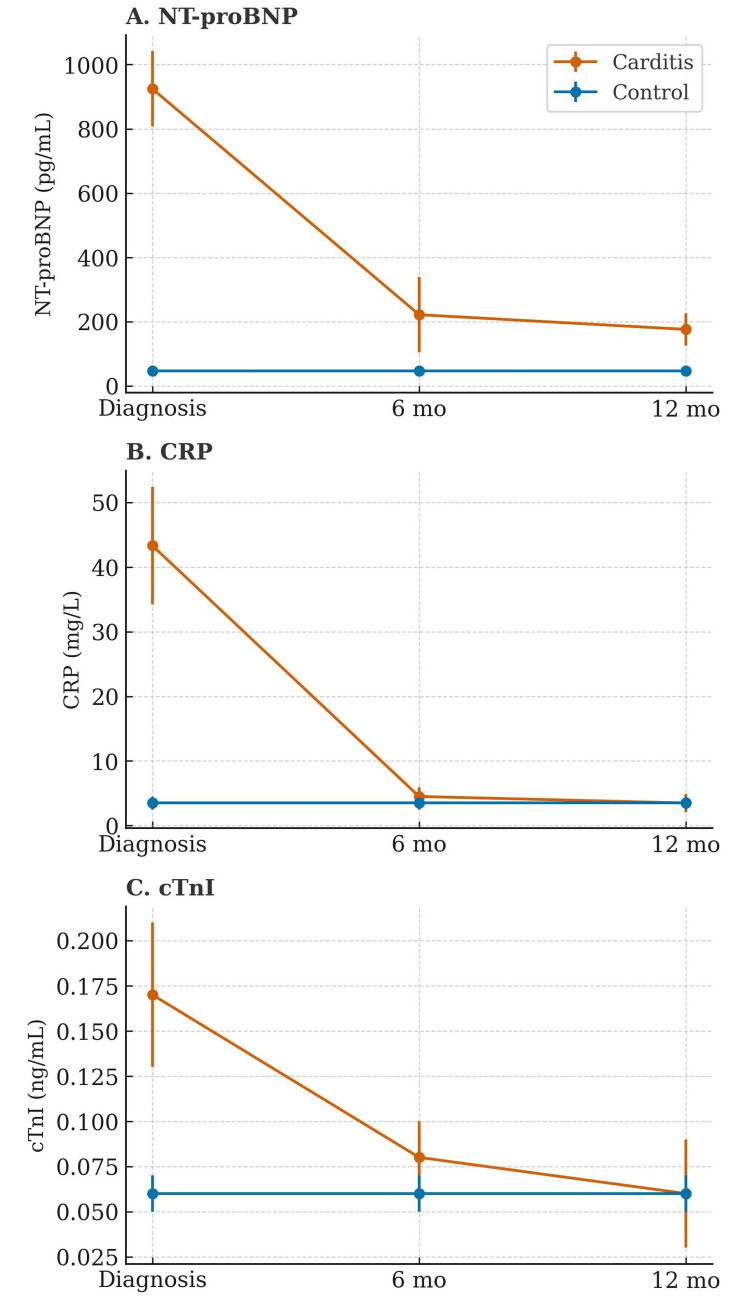
Longitudinal changes in biomarkers in children with carditis versus healthy controls. Panels show (A) NT-proBNP, (B) CRP, and (C) cTnI at diagnosis, six months, and 12 months. Values are mean ± SD. Orange: carditis; blue: controls. Abbreviations: NT-proBNP = N-terminal pro-B-type natriuretic peptide; cTnI = cardiac troponin I; CRP = C-reactive protein.

At diagnosis, NT-proBNP, CRP, and cTnI concentrations were significantly higher in the carditis group than in healthy controls (p<0.001). There was a marked decline across time for all three biomarkers (time effect p<0.001). By six months, NT-proBNP and cTnI had decreased substantially, and CRP was near normal. At 12 months, CRP had fully normalized (p>0.05 vs. controls) and cTnI approximated control values (p>0.05), whereas NT-proBNP remained modestly elevated in the carditis group (p<0.05) (Figure 2).

**Figure 3:**
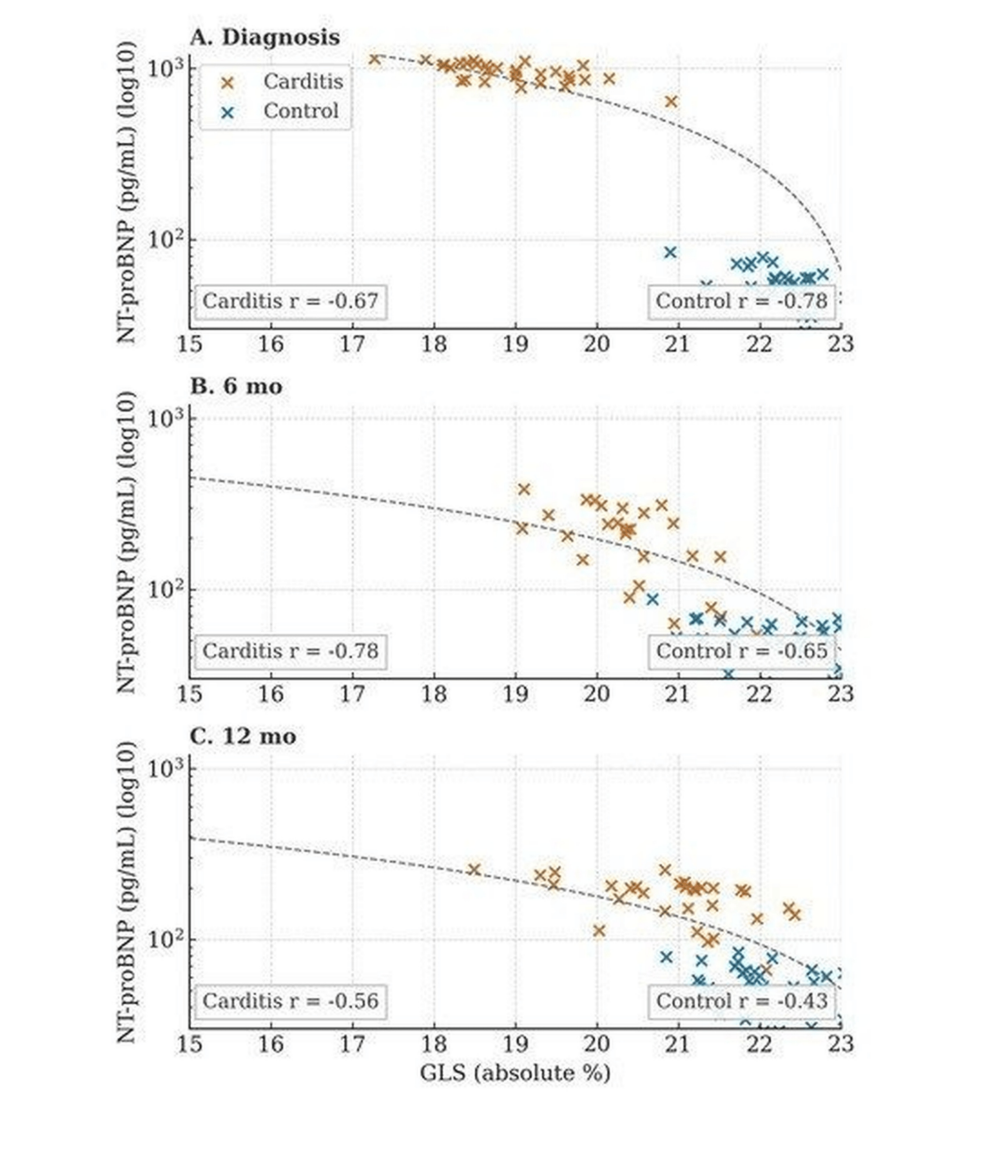
Relationship between global longitudinal strain (GLS, absolute, %) and NT-proBNP (log10-transformed) at baseline, six months, and 12 months in children with acute rheumatic carditis and healthy controls. Red points represent the carditis group; blue points represent controls. The dashed line shows the pooled linear regression fit. Small inset boxes display group-specific Pearson correlation coefficients (r) at each time point. In the pooled analysis across all participants, GLS was strongly and inversely correlated with NT-proBNP (r = –0.62, p = 0.012). Although a weak negative trend was also observed in controls, only the correlation in the carditis group reached statistical significance.

In severity sub-analyses, children with lower GLS values showed higher baseline NT-proBNP and a tendency to persistently higher levels during follow-up (group × time interaction p<0.05) (Figure 3).

Multivariable linear regression identified several variables associated with GLS: age (r = 0.42, p = 0.04), BMI (r = 0.28, p = 0.03), valve regurgitation severity (r = -0.53, p = 0.02), and NT-proBNP (r = -0.62, p = 0.012).

All participants remained on secondary prophylaxis during the 12-month follow-up. No deaths or surgical interventions occurred. Five patients with initial moderate MR improved to mild MR, but none achieved complete resolution of valve insufficiency.

## Discussion

This prospective study evaluated subclinical myocardial dysfunction in children with ARF-related carditis using GLS. Despite preserved systolic function by conventional echocardiography, GLS was significantly impaired during the acute phase, indicating early myocardial involvement that may remain undetected by standard indices.

Over the 12-month follow-up, GLS progressively improved in most patients; however, recovery was incomplete in those with moderate MR, whereas children with mild MR showed near-normalization. This suggests partial reversibility of myocardial strain depending on valvular severity.

Previous studies have similarly demonstrated subclinical myocardial involvement in RHD. Myocardial inflammation is considered a key contributor to dysfunction [14], and recent speckle-tracking studies reported reduced GLS despite preserved EF [15,16]. However, most prior investigations were cross-sectional and limited in duration. Pamuk et al. evaluated GLS before and after therapy but lacked long-term follow-up and lesion stratification [7]. Sobhy et al. combined echocardiography and cardiac MRI, showing largely reversible changes, although few patients had active carditis or significant regurgitation [17]. Our study adds novel longitudinal data with valvular severity stratification and biomarker correlations, addressing these gaps. Vasan et al. highlighted that subclinical involvement in ARF may persist for months despite apparent recovery [8]. Consistent with this, our cohort showed no severe valve deterioration, likely reflecting early detection and treatment, yet mild residual regurgitation persisted in some children, supporting the need for long-term follow-up with strain imaging.

The incomplete GLS recovery in moderate MR likely reflects sustained low-grade inflammation, early fibrosis, and chronic volume overload, consistent with histopathologic findings of persistent myocardial injury despite adequate therapy. A strong inverse relationship between GLS and NT-proBNP further underscores GLS as a sensitive marker of myocardial stress. Elevated cardiac biomarkers, particularly NT-proBNP, may account for residual strain abnormalities. Similarly, Ozdemir et al. demonstrated elevated troponin levels in active carditis, supporting the presence of subclinical myocardial injury [18]. The parallel improvement in both strain and biomarkers strengthens this physiological link.

Combined use of GLS and biomarkers may therefore enhance patient monitoring. Persistent elevation of NT-proBNP together with reduced GLS could identify children at risk of ongoing dysfunction, guiding more frequent assessments or adjunctive treatment. In resource-limited settings, biomarker trends may serve as an interim tool for identifying patients who warrant imaging follow-up.

The mechanisms underlying impaired GLS in ARF-related carditis are likely multifactorial. Although inflammation-induced myocardial injury and volume overload secondary to MR may both contribute, distinguishing the dominant mechanism is challenging in the absence of direct tissue characterization. Elevated inflammatory markers and NT-proBNP levels in our study support subclinical myocardial stress; however, cardiac MRI or T1/T2 mapping, which could differentiate edema, fibrosis, and loading conditions, was not available. Therefore, strain abnormalities should be interpreted as a combination of inflammatory and hemodynamic effects rather than a single isolated pathway. Future studies integrating advanced imaging modalities and strain analysis would help clarify this pathophysiological overlap.

Finally, the external validity of our findings should be considered. This was a single-center study involving only children with mild to moderate MR, and patients with severe MR or recurrent ARF were excluded. As a result, the results may not be fully generalizable to populations with severe valvular disease, chronic RHD, different ethnic backgrounds, or limited access to healthcare. Larger multicenter longitudinal studies are needed to confirm these results and better define which patient subgroups would benefit most from strain-guided follow-up.

Clinically, MR severity appears pivotal for follow-up intensity. Patients with moderate MR may benefit from echocardiographic evaluation every three to six months in the first year, whereas those with mild MR and normalized GLS can be followed annually. Integration of serial GLS and NT-proBNP monitoring may refine risk-adapted management and improve outcomes.

Limitations

This study has certain limitations. First, the sample size was relatively small and derived from a single tertiary pediatric cardiology center, which may limit the generalizability of the findings. Although an a priori power analysis was performed, the study should still be considered exploratory, and larger multicenter cohorts are needed to validate these results. Second, patients with severe MR were excluded to avoid the confounding effects of significant volume overload and unstable hemodynamics on strain measurements; therefore, the conclusions may not be applicable to this subgroup. Third, although strain analyses were conducted by a single experienced and blinded observer to minimize variability, inter-observer reproducibility was not assessed. Fourth, although a linear mixed-effects model with REML estimation was used to appropriately address repeated measures and missing data, random-slope modeling was not implemented; this methodological constraint should be taken into account when interpreting the results. Finally, cardiac MRI, the reference standard for detecting myocardial inflammation and fibrosis, was not performed; thus, strain abnormalities could not be validated against an advanced imaging modality.

## Conclusions

In conclusion, children with ARF-related carditis show early subclinical myocardial dysfunction detectable by GLS, despite preserved systolic function. Although most patients demonstrated recovery during follow-up, moderate MR was associated with incomplete improvement. Larger multicenter and possibly randomized controlled studies are warranted to confirm these observations and determine whether strain-guided management can improve long-term outcomes.
